# Overfishing of Small Pelagic Fishes Increases Trophic Overlap between Immature and Mature Striped Dolphins in the Mediterranean Sea

**DOI:** 10.1371/journal.pone.0024554

**Published:** 2011-09-15

**Authors:** Encarna Gómez-Campos, Assumpció Borrell, Luis Cardona, Jaume Forcada, Alex Aguilar

**Affiliations:** 1 Department of Animal Biology-Vertebrates, Institute of Biodiversity Research, Faculty of Biology, University of Barcelona, Barcelona, Spain; 2 British Antarctic Survey, Natural Environment Research Council, Cambridge, United Kingdom; National Institute of Water & Atmospheric Research, New Zealand

## Abstract

The interactions among diet, ecology, physiology, and biochemistry affect N and C stable isotope signatures in animal tissues. Here, we examined if ecological segregation among animals in relation to sex and age existed by analyzing the signatures of δ^15^N and δ^13^C in the muscle of Western Mediterranean striped dolphins. Moreover, we used a Bayesian mixing model to study diet composition and investigated potential dietary changes over the last two decades in this population. For this, we compared isotope signatures in samples of stranded dolphins obtained during two epizootic events occurring in 1990 and 2007–2008. Mean δ^13^C values for females and males were not significantly different, but age-related variation indicated δ^13^C enrichment in both sexes, suggesting that females and males most likely fed in the same general areas, increasing their consumption of benthic prey with age. Enrichment of δ^15^N was only observed in females, suggesting a preference for larger or higher trophic level prey than males, which could reflect different nutritional requirements. δ^13^C values showed no temporal variation, although the mean δ^15^N signature decreased from 1990 to 2007–2008, which could indicate a dietary shift in the striped dolphin over the last two decades. The results of SIAR indicated that in 1990, hake and sardine together contributed to 60% on the diet of immature striped dolphins, and close to 90% for mature striped dolphins. Conversely, the diet of both groups in 2007–2008 was more diverse, as hake and sardine contributed to less than 40% of the entire diet. These results suggest a dietary change that was possibly related to changes in food availability, which is consistent with the depletion of sardine stocks by fishing.

## Introduction

The exploitation of marine ecosystems is causing the rapid depletion of top predators worldwide [1], [2], [3], and small cetaceans are not an exception. This is often attributed to unsustainable, incidental bycatch rather than direct exploitation [4], [5], [6], although the depletion of food resources due to overfishing has increased [7], [8], and could be a major factor in this ecological problem.

Mediterranean marine resources have been exploited by humans for a long time and are still intensely exploited [9], [10]. As a result, the stocks of some commercial small schooling fishes have declined dramatically in the past two decades [11] and at least in western Greece, the precipitous decline of shortbeak common dolphins (*Delphinus delphis*) has been attributed to the depletion of small schooling fishes [8].

The striped dolphin, *Stenella coeruleoalba*, a cosmopolitan cetacean occurring in tropical and temperate pelagic waters, is the most abundant dolphin species in the central-western Mediterranean [12], [13]. Pollution by organochlorine compounds was the main threat for this species in the region through the second half of the 20_th_ century [14], [15] but pollutant levels are now decreasing and are thought to not represent a major threat for the species in this region [16], [17]. Furthermore, the population has probably recovered well from the epizootic that decimated it in 1990 [18] and the recent 2007 epizootic is likely to have had a much lower impact on the population [17].

Interactions with fisheries have not been considered a major threat for striped dolphins in the Mediterranean, as they occur mainly in off-shore areas of high productivity [18], [19], [20], [21], [22], [23]. Nevertheless, striped dolphins forage mainly at the shelf-break, as revealed by acoustic surveys [20], and consume a large amount of commercial fishes, as revealed by stomach content analysis [24], [25], [26]. This suggests a possible increase in competition with commercial fisheries, and the possibility of a general decrease in food availability for striped dolphins given the reduction of fish stocks over the last 20 years. This would be expected unless the dolphins had increased the consumption of cephalopods, most of which are seldom caught incidentally by commercial fisheries, with the exception of *Todarodes sagittatus* [27].

Studies on the diet of the striped dolphin around the world are traditionally based on stomach content analysis [24], [25], [26], [28], [29], [30], [31], [32], [33], [34] *inter alia*. These studies indicate that the striped dolphin is an opportunistic and generalist top predator that consumes a wide variety of pelagic and bathypelagic oceanic prey throughout the water column.

Data from stomach contents and scats from marine mammals provide crucial information on general foraging preferences, but usually produce partial or biased results on prey preference and on diet composition [35], [36]. A common problem is the over or underestimate of specific prey consumption because of unaccounted variation in different prey digestion rates [37], [38], [39], [40], [41], [42]. Furthermore, secondary ingestion of prey (ingestion of digestive tract contents of ingested prey) also leads to biased prey assessments e.g. [43]. Finally, the stomach content analysis produces only a snapshot of the overall diet, and long-term studies on feeding habits are rarely conducted. Therefore, it is difficult to accurately assess the importance of individual prey species, prey preference, and dietary shifts using stomach content analysis, although the combination of this method with stable isotope analysis has become a more effective way to reconstruct the diet of studied marine mammal populations [44], [45], [46], [47], [48], [49].

In the last few decades, stable isotope ratios of carbon and nitrogen have increasingly been applied to studies of diet to investigate the trophic relationship between species and have been successfully applied to several cetacean species e.g. [50], [51], [52], [53], [54], [55]. These studies are based on the assumption that the isotopic composition in animal tissues is related to the composition of their food resources in a predictable manner [56], [57]. Typically, the δ^13^C is related to ecological divisions within aquatic systems; inshore sources tend to be δ^13^C enriched compared to offshore sources and benthic or coastal sources show higher δ^13^C values than pelagic sources [58], [59]. δ^15^N increases between 2 and 4‰ at each trophic level, depending on the species [60]. These differences and relationships allow us to establish the differential use of habitat and resources between and within species that belong to the same food webs e.g. [45], [61], [62], [63].

Similarly, seasonal or long-term variation in diet has been addressed through isotopic signatures in a wide range of marine vertebrate species e.g. [64], [65], [66], [67], [68], [69], although not in the striped dolphin, despite the existence of numerous dietary studies. In these long-lived upper-trophic-level predators, studies of long-term variation in food resources are useful for understanding the ecological consequences of environmental change [70], since variation on consumers' diet over time almost certainly reflects changes in prey community composition.

In this study, we analysed variation in δ^15^N and δ^13^C signatures in the muscle of striped dolphins from the Western Mediterranean to: i) establish differences in the patterns of habitat use related to sex and age, ii) infer diet composition, and iii) assess temporal changes in diet over the last two decades.

## Materials and Methods

**Figure 1:**
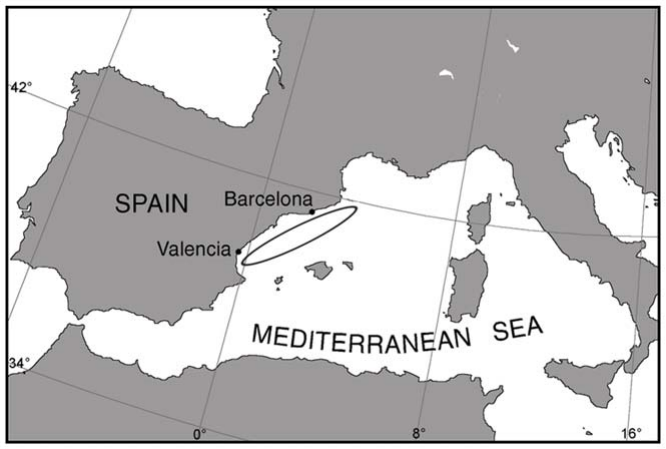
Geographical location of the study.

We sampled muscle and teeth from 116 stranded striped dolphins (61 females and 55 males) from the western Mediterranean (Figure 1) between 1987 and 2010. The specimens were collected and supplied to the authors by the Marine Animals Recovery Center (CRAM), the organism officially designated by the Catalonian regional government to collect stranded marine animals, undertake necropsies and distribute samples among research groups. Most of these samples were collected during the morbillivirus epizootics that affected the species in 1990 (n = 73) [12] and 2007–2008 (n = 17) [71], while the remainder originated from strandings not associated to these events.

Dolphin morbillivirus (DMV) causes lesions that are predominantly located in the lungs, lymph nodes, and nervous system, but metabolic changes affecting isotopic signatures have not been described [72]. Given the short time between infection and death (12–22 days in inoculated ferrets *Mustela putorius furo*, [73]; data not available for cetaceans), and the fact that the isotopic signature of the muscle reflects what the animal fed on at least 5 weeks prior to death [74], [75], the isotopic profiles analyzed in this work were not expected to be altered by the morbillivirus infection.

To minimise post-mortem degradation of tissues, only animals with a Smithsonian Institute code of 1 (live stranded and died naturally or by euthanasia) or 2 (freshly dead) [76] were considered. Muscle samples were preserved at −20°C until the analyses were performed.

The teeth were collected from the centre of the lower right jaw and prepared for age determination. The ages of the animals analysed for the current study were previously determined by Calzada et al. [77]. Briefly, the Growth Layer Groups (GLG) in teeth dentine were counted, assuming that successive pairs of lightly and darkly stained layers corresponded to a countable yearly unit. When the age of the animals were not available, standard length (cm) was used as a proxy of age. The samples covered individuals from 2 to 35 years, including immature (n = 38) and mature (n = 78) animals. Calves were excluded from the analyses to avoid confounding effects of lactation on isotope signature variation [78].

Samples of muscle from nine main potential prey species of Mediterranean striped dolphins from the study area (according to [26], [29], [79], [80]) were collected to determine their isotopic signatures. The samples were provided by local fishermen or acquired at the local market. Pelagic potential prey species included sardine (*Sardina pilchardus*), hake (*Merluccius merluccius*), blue whiting (*Micromesistius poutassou*), bogue (*Boops boops*), anchovy (*Engraulis encrasicolus*), lanternfish (*Lampanyctus crocodilus*), common European squid (*Loligo vulgaris*), and European flying squid (*Todarodes sagittatus*).

### Stable isotope analysis and Bayesian mixing model

Approximately 1 g of muscle from the dolphins and potential prey species were sampled, dried for 48 h at 60°C, and then ground with mortar and pestle. Since lipids can bias the analyses by decreasing δ^13^C levels [81], they were removed from the samples using a sequential soak in a chloroform∶methanol (2∶1) solution and shaken with a rotator to accelerate the lipid extraction. The samples were then dried again for 48 h at 40°C. Approximately 0.5 mg of powdered samples was weighed into tin capsules. Isotopic analyses were carried out by elemental analysis–isotope ratio mass spectrometry (EA–IRMS) using a ThermoFinnigan Flash 1112. Analyses were performed at the Scientific- Technical Services of the University of Barcelona.

The abundances of stable isotopes, expressed in delta (δ) notation, were the relative variations of stable isotope ratios expressed as permil (‰) deviations from predefined international standards as:
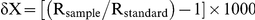
where X is ^13^C or ^15^N, and R_sample_ and R_standard_ are the ^13^C/^12^C and ^15^N/^14^N ratios in the sample and standard, respectively. The standards that were used were Vienna Pee Dee Belemnite (V-PDB) calcium carbonate for ^13^C and the atmospheric nitrogen (air) for ^15^N. International standards (IAEA) were inserted after every 12 samples to calibrate the system and compensate for any drift over time. Precision and accuracy for δ^13^C and δ^15^N measurements were 0.1% and 0.3%, respectively.

The relative contributions of potential prey species to the diet of striped dolphins were calculated with a Bayesian mixing model Stable Isotope Analysis in R SIAR) [82] package for software R [83], which took into account the isotopic signatures, elemental concentrations, and fractionation factors, and carried over the uncertainty of these values throughout the modelling process. This provides considerably more robust results than previous described models [82], [84], [85], [86]. To fit the mixing models, the isotopic values for prey species were adjusted by appropriate fractionation factors [87] obtained from the literature: 2.4 and 1.3% for δ^15^N and δ^13^C, respectively [88].

### Data analysis

Data were analysed using a Kolmogorov-Smirnov test to assess normality. The homogeneity of variances between sample groups was tested with the Levene test.

Simple linear models (ANOVA with Tukey tests for multiple comparisons and linear regressions) with fixed effects for age, size, and sex as well as mean δ^15^N and δ^13^C as the response were fitted independently. Akaike's information criterion (AIC) and small sample AIC (AICc) were used for model selection with the R software.

To investigate if stable isotope signatures changed over time, we considered two distinct periods given the sparseness of the data: the epizootic events of 1990 and 2007–2008. Since the sample size from the period 2007–2008 was relatively small (n = 17), we investigated if potential differences in isotope signatures between years could be caused by differences in age and size between years. We did not have estimates of age for the period 2007–2008, but we used size as an indicator of age. Thus, we fitted predictive Gompertz models of size (standard length) at age for females 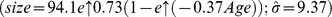 and males 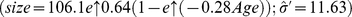 and then used model predictions of the range of variation in size given age to discuss age comparisons between periods.

Results are presented as mean ± SE. The likely contribution of potencial prey species to the diet is reported as the mean with a 95% credible interval.

## Results

### Variation in δ^15^N and δ^13^C with sex, age, and size

**Figure 2:**
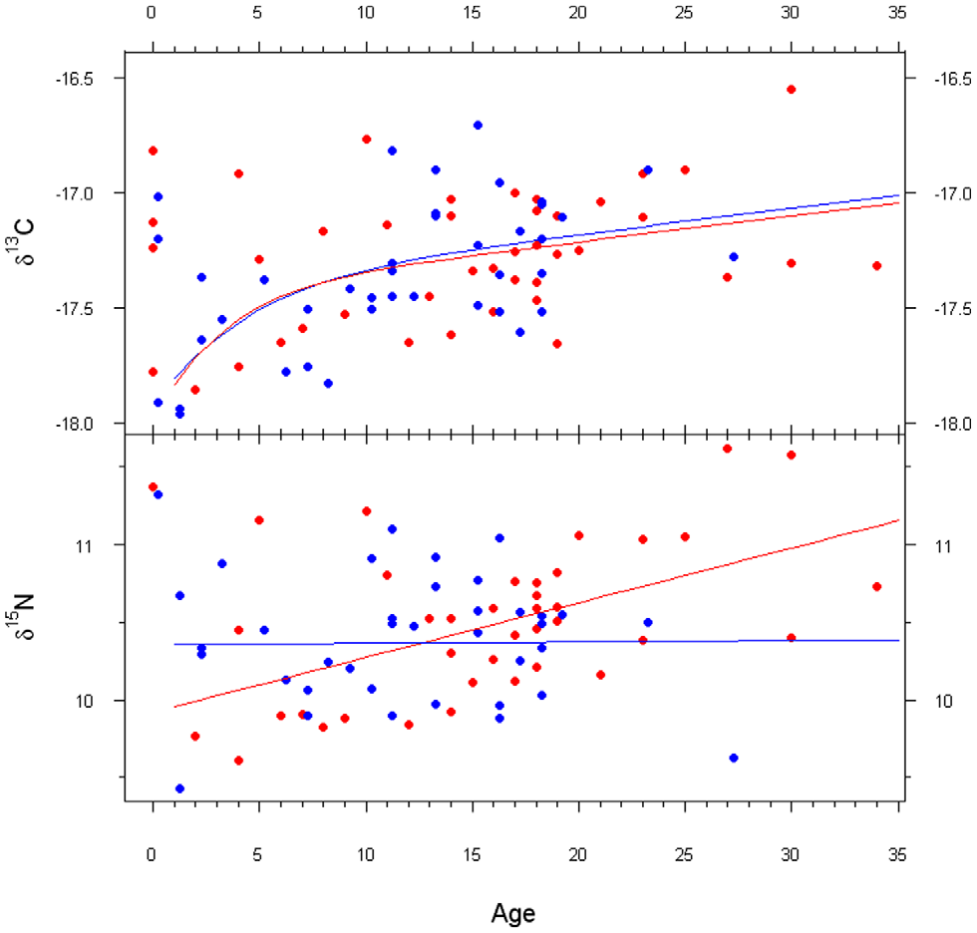
Variation of stable isotope signature according to sex and age in the Western Mediterranean striped dolphins. Lines and symbols are red for females and blue for males.

The best linear models indicated significant effects of age and size on δ^13^C (p<0.02; Table 1) without significant covariation with sex for either variable. For δ^15^N, significant effects of age, size, and sex were detected (p<0.02; Table 2). However, the slope of the fitted line for males was not significantly different from 0, while in females it was positive (Figure 2).

**Table 1:** ANOVA results for model that fit better the δ^13^C data.

	d.f.	Sum Sq	Mean Sq	F value	p
**age**	1	1.6076	1.6076	25.4118	0.0000
**Size**	1	0.3641	0.3641	5.7552	0.0190
**residuals**	72	4.5549	0.0633		

**Table 2:** ANOVA results for models that fit δ^15^N data.

	d.f.	Sum Sq	Mean Sq	F value	p
**age**	1	1.6076	1.6076	25.4118	0.0000
**size**	1	0.3641	0.3641	5.7552	0.0190
**sex∶age**	1	0.9893	0.9893	6.0209	0.0166
**residuals**	71	11.6658	0.1643		

	d.f.	Sum S1	Mean S1	F value	p
**sex**	1	0.2753	0.2753	1.6753	0.1997
**sex∶age**	1	2.6091	1.3046	7.9398	0.0008
**residuals**	71	11.6658	0.1643		

### Temporal variation in stable isotope profiles

**Figure 3:**
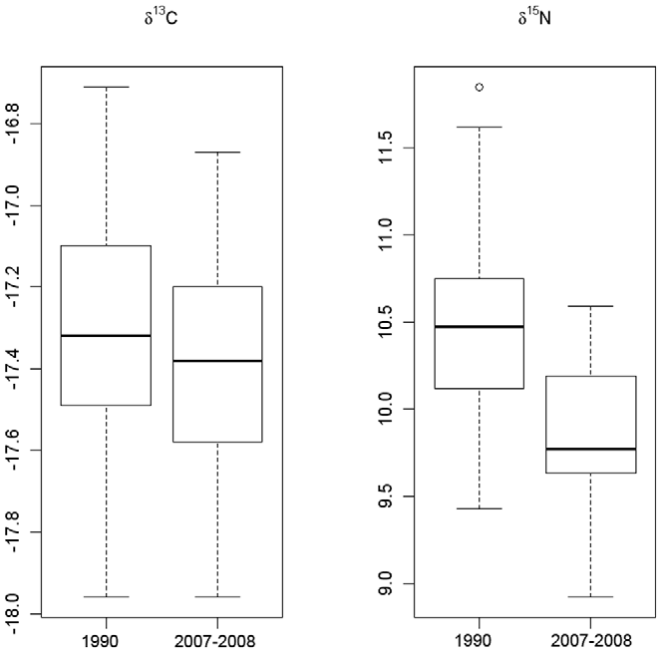
Temporal variation of δ^13^C and δ^15^N between 1990 and 2007–2008 in the western Mediterranean striped dolphins.

In 1990, the means of δ^15^N and δ^13^C in striped dolphin muscle were 10.30±0.35 and −17.37±0.25‰ respectively, whereas in 2007–2008, the means were 9.81±0.43‰ and −17.40±0.30‰, respectively. There were significant differences in δ^15^N but not δ^13^C between those periods (Table 3). The variation in isotopic signature between the two periods is shown in Figure 3.

**Table 3:** Stable isotopes values (mean±SD) measured in muscle of the potential prey and the striped dolphin.

Scientific name	Common name	n	Length (cm)	δ^13^C (‰)	δ^15^N (‰)
**Fish species**					
*ardina pilchardus*	Sardine	5	12–14	−18.0±0.2	8.7±0.2
*erluccius merluccius*	Hake	14	10–18	−19.8±0.3	8.4±0.6
*Micromesistius poutassou*	Blue whiting	5	19–33	−18.2±0.3	10.1±0.6
*Boops boops*	Bogue	10	16–21	−19.1±0.1	9.6±0.03
*Lampanyctus crocodrilus*	Lanternfish	5	6–10	−18.7±0.2	9.9±0.2
*Engraulis encrasicolus*	Anchovy	5	7–12	−18.8±0.5	9.8±0.9
**Cephalopods species**					
*Loligo vulgaris*	Common European squid	5	10–14	−17.7±0.1	9.5±0.4
*Todarodes sagittatus*	European flying squid	5	24–28	−17.8±0.1	11.1±0.1
***Stenella coeruleoalba***	Striped dolphin	116			
Immature		38	<187	−17.5±0.2	10.2±0.4
Mature		78	>187	−17.2±0.3	10.4±0.5

The mean size of females in 1990 was 190.3 cm (SD = 12.7; range: 158–208) and in 2007–2008 was 189 cm (SD = 15.5; range: 155–210), which corresponded to a mean age of 9 (range: ages 4 and above) for both years. The size was not significantly different between years. However, the δ^15^N in females (the mean for 1990 was 10.49±0.46‰, and for 2007–2008 the mean was 9.87±0.47‰) was significantly different between these years (t-test = 3.579, df = 12.1, p = 0.0037). Similarly, there was a significant difference in δ^15^N in males between the analysed periods, with a mean of 10.42±0.44‰ in 1990 and 9.74±0.4‰ in 2007–2008 (t-test = 4.312, df = 10.7, p = 0.0013). However, the sizes were not significantly different. The mean size in 1990 was 187 cm (SD = 17.84; range: 106–206) and in 2007–2008 was 185 cm (SD = 18.25; range: 148–210). In 1990, the mean age was 8 years, which ranged from 8 and above, and between 3 and above in 2007–2008. There was no significant difference between sexes for δ^15^N in 2007–2008. These results suggest that the temporal difference in δ^15^N is likely unrelated to individual sex or age differences in the two different periods.

### Mixing model and diet

**Figure 4:**
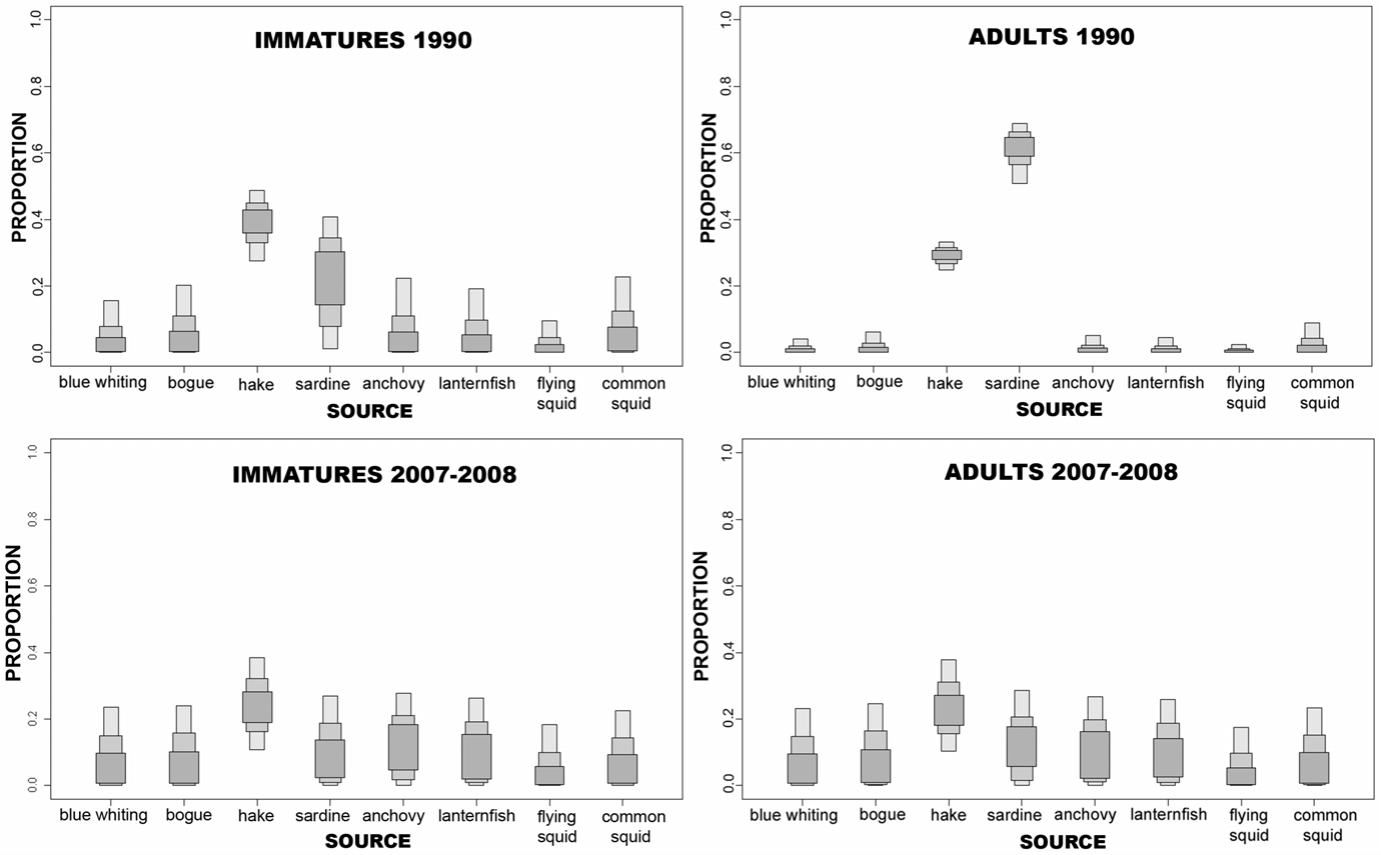
Contribution of the main potential prey to the striped dolphin diet as determined by SIAR mixing model during the 1990 (left panel) and 2007 periods (right panel) for immature and mature striped dolphins. Each prey species shows 95%, 75% and 50% credibility intervals for the calculated feasible contribution to the diet.

Table 4 shows stable isotope ratios of carbon and nitrogen in several potential preys as well as the muscle of the striped dolphin. The SIAR model was applied to the striped dolphin collected in 1990 and 2007–2008, assuming that the isotopic baseline of the western Mediterranean had not changed over the last two decades. The results of SIAR indicated that in 1990, hake and sardine together contributed to 60% on the diet of immature striped dolphins, and close to 90% for mature striped dolphins. While hake comprised a large proportion of the diet of immature dolphins (mean = 38.5%, 95% credibility interval (7.5–48.8), sardine comprised a large proportion of the diet of adults (mean = 60.3%, 95% credibility interval 50.9–68.8). Conversely, the diet of both groups in 2007–2008 was more diverse, as hake and sardine contributed to less than 40% of the entire diet. Hake was the most important diet component in both immature and adult dolphins (immature: mean = 24.2%, 95% credibility interval 10.9–38.5; adults: mean = 23.7%, 95% credibility interval 10.5–22.1), followed by sardine, which contributed to less than 15% of the total diet (Figure 4).

**Table 4:** ANOVA results for temporal differences in δ^15^N.

	d.f.	Sum Sq	Mean Sq	F value	p
**Year**	1	5.88	5.88	29.17	0.0000
**residuals**	92	18.55	0.20		

## Discussion

Sex-related differences in habitat use or feeding strategies that result in isotopic composition differences have been previously investigated in some marine mammal species, but with different and inconclusive results [53], [61], [63], [89], [90], [91], [92], [93] *inter alia*. In the present study, female and male striped dolphins showed a similar age-related enrichment in the δ^13^C signature. However, δ^15^N increased in females with age, whereas this was not detected in males.

There is no evidence of differential fractionation of stable isotopes between diet and consumer tissues between sexes [88], [94]. Therefore, the sex-related differences in isotopic signatures suggest that females and males may be segregated. It is possible that they occupy different habitats (differences in δ^13^C) and forage on different food resources (differences in δ^15^N), which could be a mechanism to avoid or reduce intraspecific competition for resources [61], [95].

The lack of differences in δ^13^C between sexes suggests that no spatial segregation in feeding occurs in Mediterranean striped dolphins. Interestingly, gender spatial segregation has been found to be more pronounced in species that show sexual dimorphism related to body size when this is a requirement to access specific food resources [61], [93]. The Mediterranean striped dolphin has no sexual dimorphism in size [96], which is in agreement with the lack of spatial segregation between sexes suggested by the similarity of δ^13^C.

On the other hand, the age-related enrichment detected in δ^13^C in both sexes could indicate an increased consumption of benthic prey with age that may be a consequence of age-related learning [93] or an increase in diving ability with physical maturation. However, this is an unlikely explanation for an off-shore species such as the striped dolphin. Phytoplankton is the sole source of organic carbon for the species inhabiting the lower shelf and the slope, and therefore pelagic and demersal species inhabiting those regions have similar δ^13^C in the western Mediterranean [97].

An alternative explanation is that immature striped dolphins had a more diverse diet than adults, as reported for the population from the northwest Pacific [28] and the north Atlantic [32], where it was found that immature striped dolphins could consume some prey depleted in δ^13^C that were not consumed by adults. This hypothesis is supported by the SIAR results, which showed that the 1990 immature dolphins had a more diverse diet than adults and included relatively large amounts of the highly depleted young hake. Conversely, adults had a narrower trophic niche and primarily consumed the highly abundant and lipid-rich sardine. These differences may be a result of the ontogeny of foraging skills and increased diving capacity progressively acquired with age.

Based on the assumption that the δ^15^N increases by approximately 3‰ for each trophic level [60], the δ^15^N enrichment with age in female striped dolphins (approximately 1‰) does not reflect an entire trophic level, but rather a significant variation in diet. This δ^15^N enrichment may be attributed to females supplementing their diet with different prey, especially through the addition of larger prey or, alternatively, foraging on different prey species than males. This could be explained by the existence of different nutritional and energetic requirements associated with reproduction in females. Changes in diet that are related to female reproduction have been described in some species of odontocetes [98], [99]. Similarly, Das et al. [53] reported that female harbour porpoises had higher δ^15^N values than males, and inferred a dietary shift related to reproduction in females. In the northeast Atlantic, some dietary differences with sex have been documented for striped dolphins through stomach content analyses; however, these differences have been poorly described and discussed [32], [34].

In contrast to previous results [24], [25], [26], [29], our analysis showed that cephalopods contribute little to the diet of the Mediterranean striped dolphin regardless of the period considered. The greater presence of cephalopods remains in the stomach content as well as the concurrent overestimate of them as a main prey in the diet is likely to be related to the digestion rates and passage times of cephalopods beaks compared to other preys.

Although no crustacean samples were available for the present study, the isotopic values for this group obtained from the literature [97] are very similar or higher in some cases than those of the striped dolphin. This may suggest that crustaceans might not be as important in the diet of the striped dolphin as it has been suggested in other studies [25], [114]. As in the case of cephalopods, the importance of crustaceans in stomach content analyses could have been overestimated because of the persistence of carapace remains in the digestive system. However, this hypothesis remains to be tested.

Temporal variation in stable isotope ratios was detected when signatures of animals from 1990 and 2007–2008 periods were compared. Over a period of almost 20 years, δ^15^N significantly decreased by approximately 1‰ on average, but no variation in δ^13^C was detected for the same period. Temporal differences in δ^15^N were unlikely to be related to sex or age differences in the animals from the two periods considered, which was shown in the current results. Similar trends have been documented in some marine mammals and fish species. In the harbour porpoise from the North Sea, δ^15^N decreased with time concomitantly with an increase in δ^13^C, which were changes that the authors attributed to the consumption of smaller prey [92]. In contrast, the δ^13^C of South American sea lions (*Otaria flavescens*) increased in northern Patagonia from the 1940s to the 1970s and then declined, whereas the δ^15^N was unaffected due to changes in prey availability that were caused by industrial fishing [93]. Wainright et al. [100] found a significant decline in δ^15^N in haddock (*Melanogrammus aeglefinus*) between 1929 and 1987 in the Georges Bank off of the coast of Maine, and suggested that the observed decline may indicate a collapse in trophic structure towards a simpler food web with fewer trophic levels.

In the present study, it is assumed that the stable isotope baselines did not change, which is in agreement with available evidence that indicates no major changes in the productivity of the western Mediterranean [101]. If this is true, the observed trends in δ^15^N can only be explained by a dietary shift with a reduced consumption of sardine by adult striped dolphins in 2007–2008. This shift is consistent with the changes in the structure of the ecosystem reported for the study area during the past three decades, including a decrease in the abundance of sardine and the stability in the abundance of young hake [11], [102]. Sardine is one of the most important species in terms of both biomass and commercial interest in the western Mediterranean [11]. This species, similar to other small pelagic fish populations, is subject to considerable fluctuations caused by environmental variability [103] and intense commercial exploitation. The commercial exploitation of small schooling fish in the northwest Mediterranean has been significant since the early 1940s [104]. The sardine biomass initially showed a clear and important increase from 1978 to the mid 1990s, but has decreased since the mid 1990s [11], [102], [105]. On the other hand, hake populations are fully exploited or overexploited [106], [107], [108], which has resulted in a decreased biomass of large hake and increased biomass of small hake [102], [109]. The decrease in the adult hake biomass seems to be mainly due to an increase in the long line fishing effort. Moreover, the steady and intense decline of anglerfish and other demersal predators, including adult hake, would have increased the juvenile hake population due to a lack of predation. These declines in the biomass of predatory fish might have caused an increase in the biomass of other organisms, such as benthic invertebrates and benthopelagic fish, which are also prey for juvenile hake [102], [110].

Given the progressive decline in sardine abundance since the mid 1990s and the parallel increase in juvenile hake, the dietary shift reported here for the Mediterranean striped dolphin could respond to prey availability during each period, according to the general and opportunistic feeding behavior that is widely described around the world. It is worth to note that juvenile hake was not the only species whose consumption by adult striped dolphins increased between 1990 to 2007–2008. This was also true for the European flying squid, anchovy and lanternfish and according to models developed by Coll et al. [9], the proliferation of certain species in low trophic levels (shrimps and benthic invertebrates) or with higher turnover rates (cephalopods and benthopelagic fish) would be compatible with the decrease in biomass of higher trophic level fish and small pelagic fish observed in the Catalan Sea. Similar changes have been previously documented in other Mediterranean areas [111], [112], [113], *inter alia*. Therefore, the increase in consumption of cephalopods, anchovy and lanternfish during the last period could be related to a higher availability of these resources, at a time when the abundance of sardine decreases due to overfishing. This change in diet would explain the observed differences in δ^15^N between the two periods and the lack of differences in δ^13^C, as both sardine and juvenile hake are pelagic species occurring primarily over the continental shelf.

Such a change, from a sardine-dominated diet to a hake-dominated one might have some negative long term impacts on the population of striped dolphin in the Catalan Sea, as sardines have a much higher fat contents and energy density than hake [115]. Although the increased consumption of lipid-rich anchovies and lanternfish may have partially compensated for the reduced consumption of sardines by adult striped dolphins, a lower energy intake can result in a host of physiological effects that can ultimately impact in immediately and future life history parameters, such as deficient body composition, inadequate energy budgets, lower growth rates, changes in reproductive cycles and various physiological disorders [116], [117].

The amount of food required depends of both energy requirements and prey quality and when prey quality diminishes, the occasioned deficit could be supported by increasing food intake. Several studies have demostrated that, in some situations, this may suffice marine mammals to maintain body mass and composition [116], [118], [119]. However, the amount of prey that an animal can capture and process during a period of time is limited by the animals' physiological capacity, and in some cases feeding *ad libitum* low-quality prey is not enough to compensate energy requirements [120], [121].

Furthermore, a low quality diet could not affect in the same manner all the stratum of the population. Individuals with higher energy and nutritional demands and lower or restricted foraging skills, such as young individuals and pregnant or lactating females, could have more difficulties to consume and process sufficient prey to meet their energy demands and becomes more vulnerable to dietary shifts. Maternal restriction during pregnancy has been associated with alterations in growth and functions of core tissues and abnormal development in calves of several mammal species [122], [123], [124], [125], [126], [127], [128], *inter alia*. Moreover, it has been demostrated that small calves subsequently produce small offspring that may be more vulnerable to environmental extremes and other causes of mortality [129], [130].

The best case study is provided by the decline of the Steller sea lion (*Eumetopias jubatus*) population, associated with a chronic decline in female reproductive rates over almost 30 years and a decreased juvenile survival [131], [132] due to a reduction of food quality [116]. This change in fecundity might result from mothers who, being unable to maintain body condition due to nutritional restrictions (due to decreased energy intake and or increased lactational demands from young who need additional supplementation), are not giving birth every year, due to either spontaneous abortions or nutrition-related anoestrous.

There is no current evidence of those changes in Mediterranean the striped dolphin population, but differences in growth patterns, such as length at birth, length at sexual maturity and maximum body size, between the Mediterranean and western Pacific populations suggest that it is sensitive to changes in per-capita food availability [133], [134]. Further investigations are needed to assess ecological implications of dietary shifts in the Western Mediterranean striped dolphin.
